# Multivariate time series data of milling processes with varying tool wear and machine tools

**DOI:** 10.1016/j.dib.2023.109574

**Published:** 2023-09-14

**Authors:** Berend Denkena, Heinrich Klemme, Tobias H. Stiehl

**Affiliations:** Institute of Production Engineering and Machine Tools, Leibniz Universität Hannover, 30823 Garbsen, Germany

**Keywords:** Machine tools, Milling, Time series, Wear, Remaining useful life, Fleet monitoring, Transfer learning

## Abstract

Machining is an essential part of modern manufacturing. During machining, the wear of cutting tools increases, eventually impairing product quality and process stability. Determining when to change a tool to avoid these consequences, while still utilizing most of a tool's lifetime is challenging, as the tool lifetime can vary by more than 100% despite constant process parameters [1]. To account for these variations, all tools are usually changed after a predefined period of time. However, this strategy wastes a significant proportion of the remaining lifetime of most tools. By monitoring the wear of tools, all tools can potentially be used until their individual end of life. Research, development, and assessment of such monitoring methods require large amounts of data. Nevertheless, only very few datasets are publicly available. The presented dataset provides labeled, multivariate time series data of milling processes with varying tool wear and for varying machine tools. The width of the flank wear land VB is used as a degradation metric. A total of nine end milling cutters were worn from an unused state to end of life (VB ≈ 150 µm) in 3-axis shoulder milling of cast iron 600–3/S. The tools were of the same model (solid carbide end milling cutter, 4 edges, coated with TiN-TiAlN) but from different batches. Experiments were conducted on three different 5-axis milling centers of a similar size. Workpieces, experimental setups, and process parameters were identical on all of the machine tools. The process forces were recorded with a dynamometer with a sample rate of 25 kHz. The force or torque of the spindle and feed drives, as well as the position control deviation of feed drives, were recorded from the machine tool controls with a sample rate of 500 Hz. The dataset holds a total of 6,418 files labeled with the wear (VB), machine tool (M), tool (T), run (R), and cumulated tool contact time (C). This data could be used to identify signal features that are sensitive to wear, to investigate methods for tool wear estimation and tool life prediction, or to examine transfer learning strategies. The data thereby facilitates research in tool condition monitoring and predictive maintenance in the domain of production technology.

Specifications Table

**Table:** 

Subject	Manufacturing Engineering
Specific subject area	Tool condition monitoring and remaining useful life prediction in milling of cast iron
Data format	Filtered multivariate time series
Type of data	.h5 (multivariate time series in HDF5 format) .csv (overview of all h5 files including labels)
Data collection	Milling experiments were conducted with three different machine tools. Numerical control instructions were identical, workpieces were from a single batch of material, and tools were of the same model, but from different batches. The process forces were recorded with a dynamometer. The force and torque of feed drives, position, and position control deviations were recorded from the machine tools’ controls. The width of the flank wear land VB of peripheral cutting edges was measured with a digital microscope at a magnification of 100x.
Data source location	Recorded and stored at the Institute of Production Engineering and Machine Tools, Leibniz Universität Hannover, 30,823 Garbsen, Germany
Data accessibility	Repository name: Mendeley Data (https://data.mendeley.com/) DOI:10.17632/zpxs87bjt8 Direct URL to data: https://data.mendeley.com/datasets/zpxs87bjt8

## 1 Value of the Data

- The data is useful as it is the first to provide the tool life trajectories for varying machine tools. In the data that is currently publicly available, only tools, process parameters, or materials are varied (e.g. [2]). Additionally, the data provided in this publication adds to the currently very limited amount of publicly available data for machining.
- The data benefits researchers and product developers as it reduces the need to conduct additional experiments, which is a task that usually consumes many resources.
- The data can be used to develop new methods for tool condition monitoring and remaining useful life prediction. In addition, the data enables different methods to be benchmarked. The data might also be used to identify similarities and dissimilarities between multiple machine tools or to investigate transfer learning applications.

## 2 Data Description

**Fig. 1:**
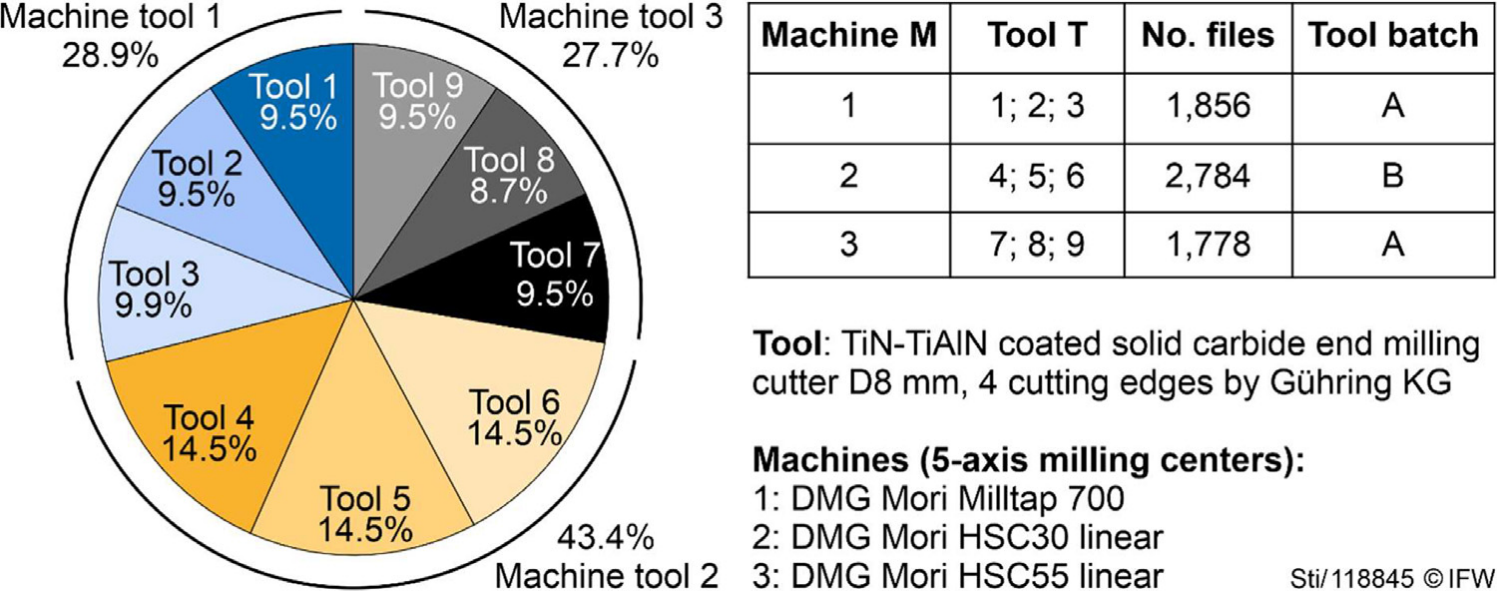
Share of sample files by machine tools and tools.

The dataset holds a total of 6418 files with individual samples and a file titled “filelist.csv”. The file “filelist.csv” provides an overview of all the sample files and their corresponding labels. Sample files are in the .h5 format (HDF5). A total of nine tools were worn from an unused state (VB ≈ 0 µm) to their end of life (VB ≈ 150 µm). The number of experimental runs (sample files) varies per tool. Fig. 1 depicts the share of sample files subdivided into machine tools and tools.

Table 1 describes the labels and their identifiers. The labels are saved within each sample file and are additionally coded into the filename. For example, the file “M2T6R30C170VB45.h5” was recorded on machine tool M2 with tool T6, it is the 30th experimental run of the tool with a cumulated tool contact time of 170 s at the beginning of the run, and has a wear label of VB = 45 µm.

**Table 1:** Description of labels.

Identifier	Label	Description	Unit
M	Machine	Number of machine tool	–
T	Tool	Number of tool	–
R	Run	Counter of experimental runs recorded for each tool; Only successfully recorded runs are counted; A run is a single shoulder milling operation	–
C	Cumulated tool contact time	Time a tool has actually been machining material before the current run	s
VB	Wear	The width of the flank wear land VB of the tool	µm

It is recommended to use the label “wear” (VB) as the degradation metric and the label “run” (R) or “cumulated tool contact time” (C) as the degradation trajectory. A run is a single shoulder milling operation. The label “run” provides the order in which runs were recorded, specific for each tool. However, recordings of some runs were corrupted and had to be removed. Runs were then renumbered in an incremented, continuous order. Therefore, the label “run” does not account for deleted runs. The label “cumulated tool contact time”, on the other hand, represents the actual time a tool has been used for machining. The difference between “run” and “cumulated tool contact time” can be demonstrated by the example of tool T8. The recordings of the first runs for tool T8 were corrupt and consequently removed. The first usable run already has a cumulated tool contact time of C = 177 s. This run, however, is labeled as run R = 1.

**Fig. 2:**
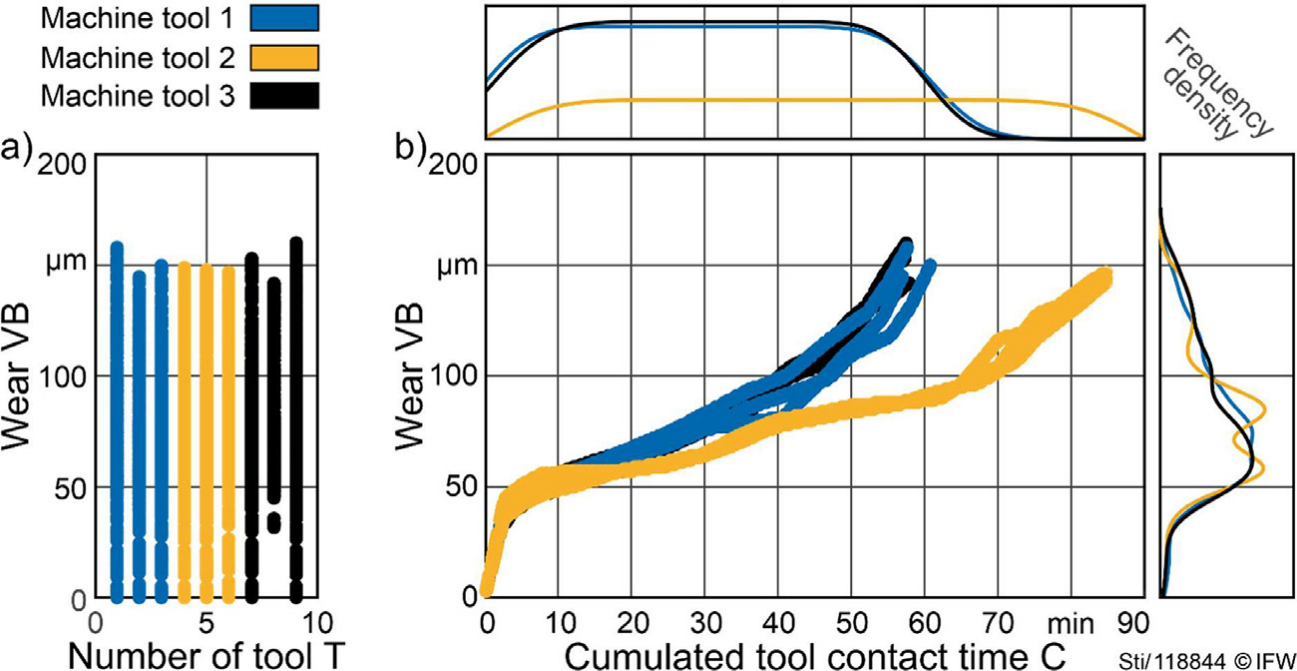
Distribution of sample files.

Fig. 2 depicts the distribution of labels for each machine tool and the degradation trajectory of individual tools. Tool life starts at VB = 0 µm and the end was defined at VB ≈ 150 µm. The first runs of tool T8 are missing (Fig. 2a; gap for tool T8 for VB < 50 µm). Tool lives, as expressed in time, varied (Fig. 2b). As depicted by the frequency density plots, the majority of runs are within a range of wear of 50 µm to 100 µm.

**Fig. 3:**
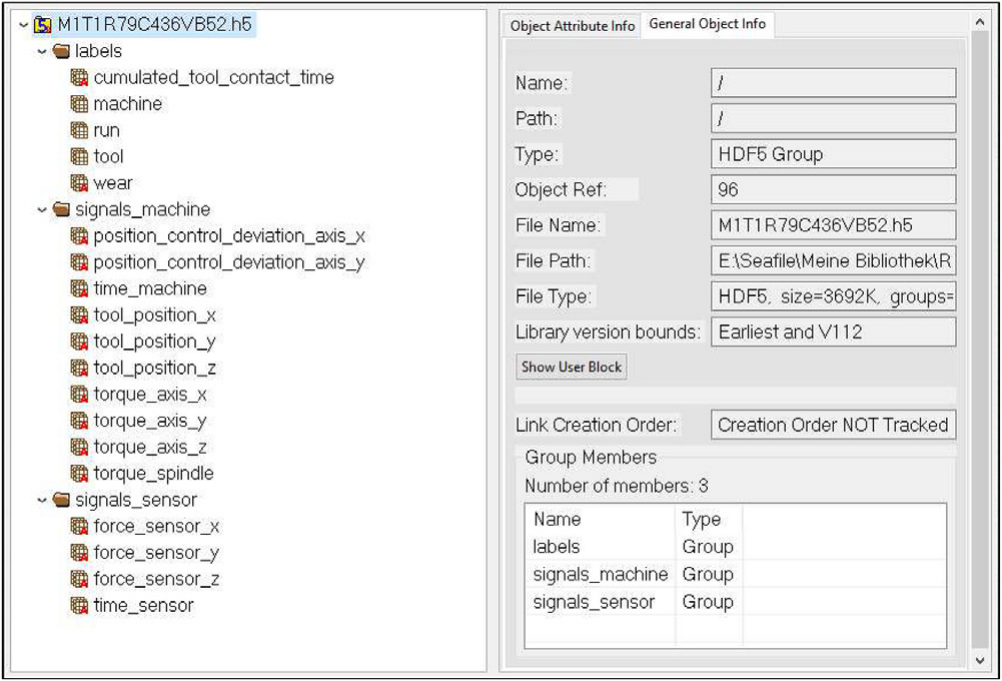
Structure of HDF5 files when opened with the software HDF view.

**Fig. 4:**
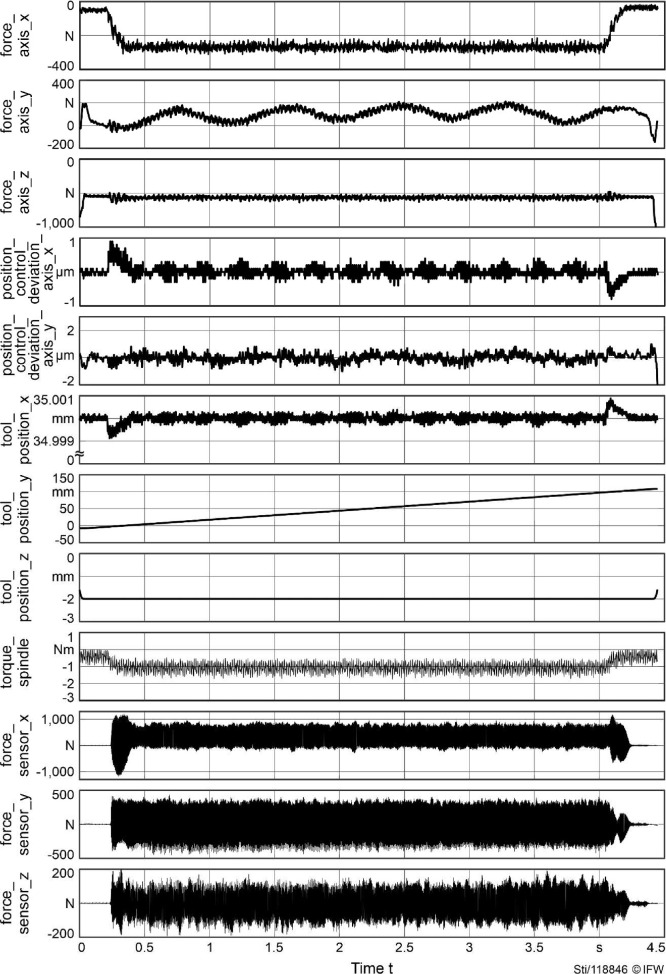
Example of typical signals recorded from machine tool M3.

The recorded signals are provided as time series and are grouped by the source they were recorded from (Table 2). Signals were either recorded from the control of the machine tool or from the dynamometer (sensor). For machine tools with linear feed drives (M2 and M3), the signal “force_axis” is provided. For machine tools with feed drives using ball screw drives (M1), the signal “torque_axis” is provided. The tool position is defined in the workpiece coordinate system. Fig. 3 depicts the structure of the provided HDF5 files. Fig. 4 gives an example of the typical signals recorded from machine tool M3.

**Table 2:** Description of time series data.

Name of signal	Unit	Source	Available for machine		
			M1	M2	M3
time_machine	s	machine	x	x	x
position_control_deviation_axis_x	µm	machine	x	x	x
position_control_deviation_axis_y	µm	machine	x	x	x
tool_position_x	mm	machine	x	x	x
tool_position_y	mm	machine	x	x	x
tool_position_z	mm	machine	x	x	x
torque_spindle	Nm	machine	x	x	x
torque_axis_x	Nm	machine	x		
torque_axis_y	Nm	machine	x		
torque_axis_z	Nm	machine	x		
force_axis_x	N	machine		x	x
force_axis_x	N	machine		x	x
force_axis_x	N	machine		x	x
time_sensor	s	sensor	x	x	x
force_sensor_x	N	sensor	x	x	x
force_sensor_y	N	sensor	x	x	x
force_sensor_z	N	sensor	x	x	x

## 3 Experimental Design, Materials and Methods

A total of nine end milling cutters were worn during shoulder milling on three different machine tools (Table 3). The tools were of the same model, but from different batches. Workpieces were from a single batch of cast iron (600–3/S according to ISO 1083). Process parameters were constant throughout all experiments and all of the machine tools used identical NC-instructions, as well as experimental setups. For the experiments of tool T8, the first runs of the initial wear phase are missing. For tools T7 and T8, the workpiece coordinate system did not match the machine coordinate system (detailed explanation after Fig. 6). This mismatch mostly affected the signals “position_axis”, “position_control_deviation_axis”, and “force_axis”.

**Table 3:** List of conducted experiments.

Tool T	Machine M	Part	Tool batch	Material batch	Comment
1	1	1	A	A	–
2	1	2	A	A	–
3	1	3	A	A	–
4	2	4	B	A	Aliasing in force_axis, torque_spindle
5	2	5	B	A	Aliasing in force_axis, torque_spindle
6	2	6	B	A	Aliasing in force_axis, torque_spindle
7	3	7	A	A	Coordinate mismatch
8	3	8	A	A	Coordinate mismatch, first runs missing
9	3	9	A	A	–

**Fig. 5:**
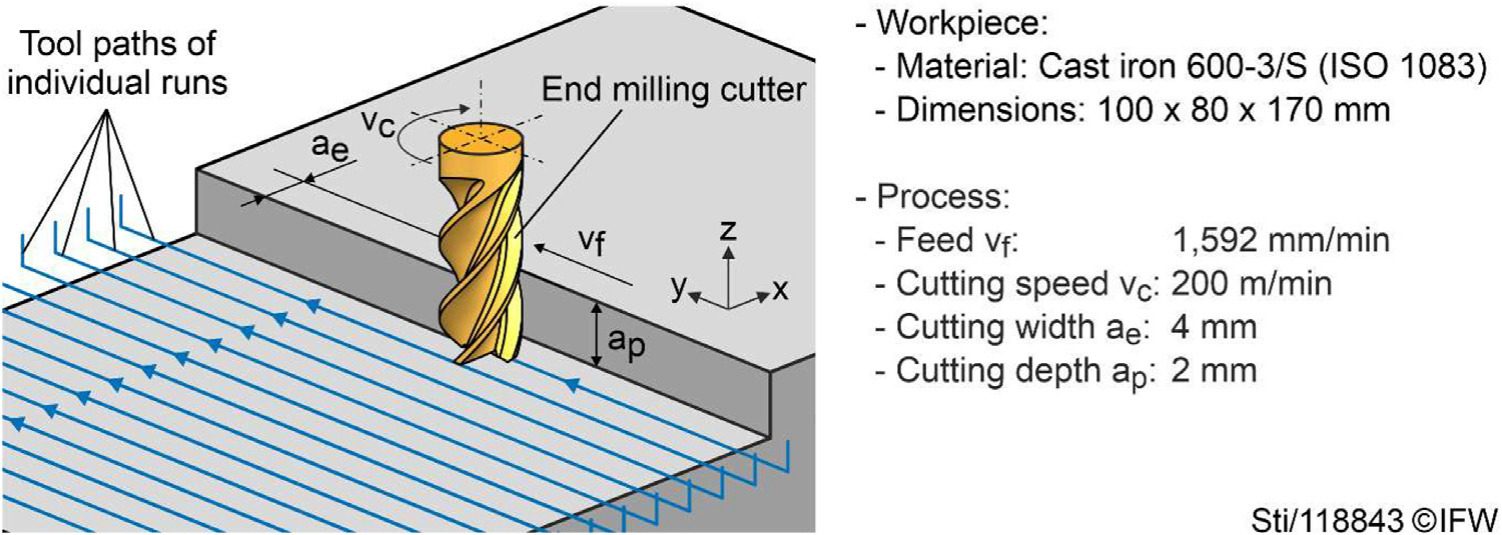
Milling process and process parameters used for all experiments.

Fig. 5 depicts the milling process and process parameters. One workpiece per tool is used to wear the tool from its unused state to its end of life. Workpieces were machined layer by layer with shoulder milling processes. Machining a single layer required 44 runs of shoulder milling operations. Each run is saved to a separate file. The depth of cut (height of a layer) was a_p_ = 2 mm. Machining continued until the tools reached a wear VB of about 150 µm.

**Fig. 6:**
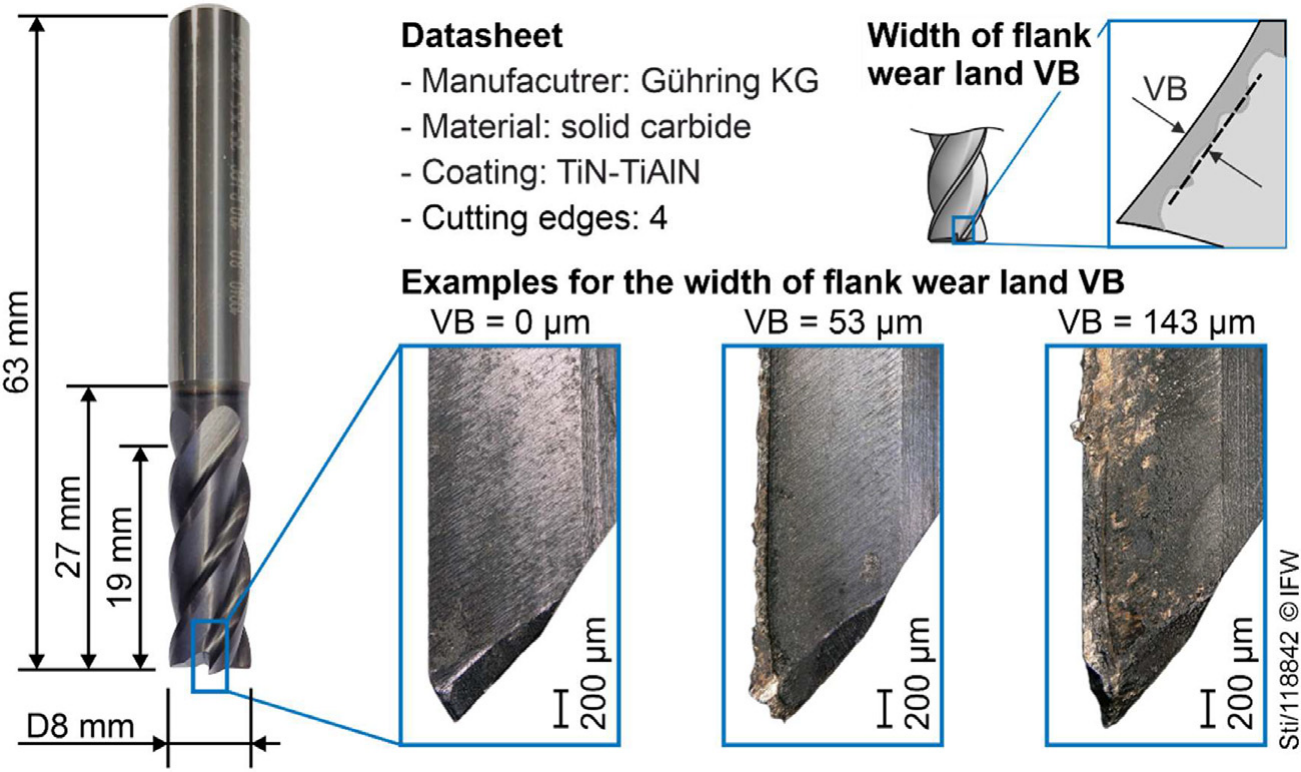
End milling cutter used and typical wear.

Wear was quantified by the width of the flank wear or the uniform flank wear VB according to ISO 8688–2 (Life testing of milling tools; Part 2: End milling). VB was measured with a digital microscope from Keyence (model VHX600DSO, 1/1.8 CCD sensor, 1600 × 1200 pixels, 100x magnification). The width of the flank wear land was measured separately for all four peripheral cutting edges of the tool. The reported width of the flank wear land VB is the average of all four cutting edges. Measurement uncertainty for the reported width of the flank wear land VB was determined to be about 5 µm. Examples of typical wear, along with the used end milling cutter and its properties, are depicted in Fig. 6. In the initial and final stages of the tool life, wear was measured after each layer of material was removed from the workpiece. During the remaining, linear phase of tool life, wear was measured after every second layer.

**Fig. 7:**
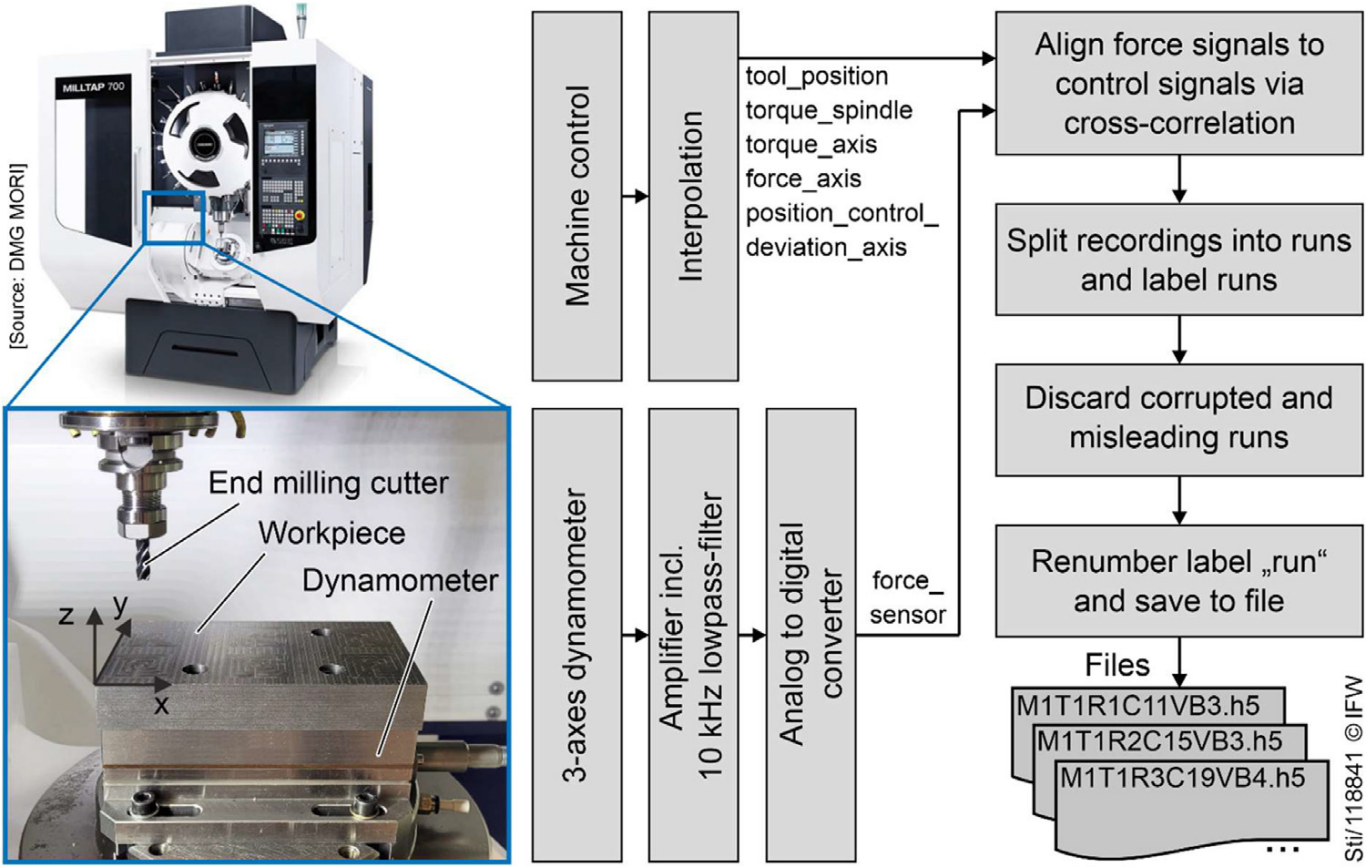
Experimental setup and processing workflow at the example of machine tool M3.

Workpieces were mounted on top of the force sensor (dynamometer). The axes of the force sensor were aligned to the coordinate system of the machine (Fig. 7). A coordinate mismatch occurred for tools T7 and T8. While the workpiece coordinate system was aligned with the dynamometer, it was not aligned with the machine tool axis. The axes X and Y were rotated by about 1° to 2° due to an active coordinate transformation rotating the XY plane. The machining process itself was executed as defined in the NC-instructions. However, for a motion purely defined in the Y-direction, the X and Y axis moved, instead of the Y-axis only.

The signals “force_sensor” were measured with a dynamometer type 9257B by Kistler Instrumente AG. Force signals were processed with charge amplifiers type 5015 by Kister Instrumente AG which included a low-pass filter with the cut of frequency f_c_ = 10 kHz. Force signals were recorded with an AD-Converter type NI 9215 by National Instruments Corp. at a sample rate of 25 kHz. All other signals were recorded from the machine tools’ controls. For machine tools with controls by Siemens AG (Sinumerik 840D sl), signals were recorded via the built-in trace function at a sample rate of 500 Hz. For machine tools with controls by Heidenhain Corp. (iTNC 530), signals were recorded via the TNCScope software by Heidenhain Corp. at a sample rate of 500 Hz. However, some controls output a varying sample rate or provide position signals at a sample rate of only 125 Hz. Therefore, signals recorded from the controls were interpolated to a uniform sample rate of 500 Hz. For the signals “tool_position”, a shape-preserving piecewise cubic interpolation was used and for all other signals sourced from the control, a linear interpolation was used.

The controls of machine tools usually apply an internal low-pass filter to certain signals when recording. For Sinumerik 840D sl controls by Siemens AG, this is typically a first-order lack acting as a low-pass filter. However, for machine M2 (DMG Mori HSC30 linear), this filter was disabled during recording. Consequently, the signals “force_axis” and “torque_spindle” are affected by aliasing (recorded signals are distorted when compared to the original continuous signal).

After the completion of experiments, the signals “force_sensor” were aligned to the signals recorded from the machine tool controls. Cross-correlation was used to find the optimal alignment between the combined force of the X and Y direction and the signal “torque_spindle”. Then, the offset of the time vector “time_sensor” was adapted accordingly. Despite this procedure, a misalignment of about 100 ms might remain in some cases. Consequently, the signals “force_sensor” should not be used to analyze the point of time of material entry or exit etc.

Wearing a tool required a series of repetitive shoulder milling operations. When recording the raw data, each recording held multiple machining operations. To make data easier to handle, the recordings were split into individual runs. Each of these runs represent a single shoulder milling operation, defined as the tool entering the material, machining a strait path, and the tool exiting the material. The individual runs were separated by analyzing the feed velocity calculated from the position signals x, y, and z following [3]. Figs 4 and 5 depict examples of the tool paths from the resulting runs.

The wear VB is measured after machining one or two entire layers of the workpiece, which translates to a measured VB label roughly every 40 runs. For all other runs, the wear label VB was interpolated with a linear function for the beginning of each run using the cumulated tool contact time. However, the wear label VB should be interpreted as an estimation of the wear condition of the entire run due to the uncertainties resulting from measurement and interpolation. After runs were fully labeled, unusable runs were discarded. This included the first and last run of each layer that was machined, runs impaired by the holes for mounting the workpiece, and runs with corrupted data. Runs were then renumbered in an incremented, continuous order for each tool.

Table 4 gives an overview of the machine tools used in the experiments. The machine tools have similar workspace dimensions, drive powers, and complexity (5-axis). It is therefore assumed that these machine tools would be used for machining similar components with similar tools. Accordingly, the group of machines is considered representative of an industrial application of transfer learning across multiple machine tools. Differences between the machines exist, for example, in terms of drive concepts and controls.

**Table 4:** Description of employed 5-axis milling centers.

Machine M	Model	Control	Feed drive	Main spindle	Machining space
1	DMG Mori Milltap 700	Siemens Sinumerik 840D sl	Ball screw	4 kW / 8 Nm	700 × 420 × 380 mm
2	DMG Mori HSC 30 linear	Siemens Sinumerik 840D sl	Direct drive	15 kW / 12 Nm	320 × 300 × 280 mm
3	DMG Mori HSC 55 linear	Heidenhain iTNC 530	Direct drive	55 kW / 19 Nm	450 × 650 × 460 mm

## 4 Limitations

- The label “run” (R) is an ordered sequence that only accounts for recorded runs. The label “cumulated tool contact time” (C) tracks the true machining time of each tool.
- All tools: The offset of the time vector for the force signals F_X_, F_Y_, and F_Z_ was modified so that the signals align with the signals recorded from the machine tool's control. Therefore, no conclusions should be drawn from comparing temporal aspects of the force signals to the signals recorded from the control. However, temporal relations between the force signals themselves were maintained and can be analyzed.
- Tools T4, T5, T6 (machine tool M2): The signals “force_axis” and “torque_spindle” might be affected by aliasing (signals include distortions and might not represent the true signal).
- Tools T7, T8 (machine tool M3): The workpiece coordinate system was rotated by about 1° to 2° in the XY-plane in reference to the machine tool axes. Due to this misalignment, a motion purely programmed in the X or Y direction will cause an unintended move of both axes, X and Y, simultaneously.
- Tool T8 (machine tool M3): The first runs, when the tool was unused, are missing.

## Ethics Statement

The data provided is original and is free of secondary data or data collected from social media platforms. Data collection did not involve any human subjects, animal experiments, chemicals, procedures or equipment that have any unusual hazards inherent in their use.

## CRediT authorship contribution statement

**Berend Denkena:** Supervision. **Heinrich Klemme:** Writing – review & editing. **Tobias H. Stiehl:** Conceptualization, Methodology, Software, Formal analysis, Data curation, Writing – original draft.

## Data Availability

Multivariate time series data of milling processes with varying tool wear and machine tools (Original data) (Mendeley Data)
